# The costs of a sexually transmitted infection outreach and treatment programme targeting most at risk youth in Tajikistan

**DOI:** 10.1186/1478-7547-7-19

**Published:** 2009-11-03

**Authors:** Nisso Kasymova, Benjamin Johns, Benusrat Sharipova

**Affiliations:** 1 YPHDP and HIV/AIDS Officer, UNICEF Dushanbe, 37/1 Bohktar Street Tajikistan; 2 Consultant, 701 Cathedral Street, Baltimore, MD 21201 USA; 3 Consultant, Dushanbe, Tajikistan

## Abstract

**Background:**

Targeted outreach, counselling, and treatment of sexually transmitted infections (STIs) are among the most cost-effective interventions aimed at ameliorating the burden of HIV/STIs. Since many new HIV infections occur in people under the age of 25, youth, and especially most at risk adolescents (MARA), need to be able to access HIV/STI services. Starting in 2006, a programme targeting MARA including outreach, confidential and voluntary counselling and testing, and STI diagnosis and treatment was piloted in three cities in Tajikistan. This study uses data from these pilot sites to estimate the costs of a national programme.

**Methods:**

Cost data were collected from the three pilot sites. Then, the target population and the number of patients receiving specific types of services are calculated for other areas. The unit costs from the pilot sites are multiplied by usage rates to determine the total costs of a national programme. Scenarios were developed to reflect data uncertainty. The government's ability to finance the programme was estimated using Ministry of Health budget data. Further analyses were done for one of the pilot cities where more detailed data were available.

**Results:**

In total, costs were projected for eight programme sites, covering an estimated 8,020 MARA. Operational and variable cost for the programme are projected to be US$ 119,159 (range US$ 104,953 to 151,524) per year. Including annual equivalent cost for capital and start-up items raises this to US$ 137,082 (range: US$ 123,022 to 169,597) per year. The analyses of potential sources of financing for the programme remain inconclusive, but it may take multiple sources of financing to fund the programme.

**Conclusion:**

While the cost-effectiveness of similar programmes have been previously assessed using modelled data, more work needs to be done to assess the costs of new programmes in relation to financial resources available. Full costing should consider cost-savings as well as expenditures. If feasible, the impact of the programme should be monitored over time.

## Background

Internationally, about 50% of HIV incidence cases are among youth aged 15 to 24, which has led to an increased concern with preventing the transmission of HIV among this age group [1]. A recent systematic review points towards the need to provide programmes that target youth, accommodate their needs, and provide assurances of confidentiality and high quality treatment [1]. Specifically, for youth most at risk of HIV infection, increased access to information and services, especially treatment of sexually transmitted infections (STIs), were recommended [1].

Targeted outreach, counselling, and treatment of STIs have been shown to be among the most cost-effective interventions in the prevention or care of sexually transmitted infections including HIV/AIDS. For example, one recent review found peer education and treatment of STIs in sex workers to be the most cost-effective of all interventions surveyed in South Asia, and the second most cost-effective (after mass media) in the highest mortality region of Africa [2]. Another review across low- and middle-income countries found peer education and outreach to be the most cost-effective and STI diagnosis and treatment the third most cost-effective of all HIV/AIDS interventions surveyed [3]. Both studies found these interventions to be very cost-effective when compared with public health interventions in general [4,5].

There remains, however, little information available on the costs of both outreach to vulnerable populations and the subsequent treatment of STIs [6], and almost no information on the costs of programmes targeting youth. The available information shows that unit costs at a particular service site vary by country and by the volume of patients [6-9]. While these cost data indicate that there is likely economies of scale, or that there is a U-shaped long-run average cost curve, in the delivery of STI treatment to commercial sex workers (CSWs), these data alone do not provide information on the cost for the expansion of programme services to areas where they were previously unavailable [6,10]. Information on the number of people to be served at new programme sites, together with data on the variables that likely cause economies of scale, are needed in order to plan for the costs of new or expanded programmes.

This paper seeks to add to the literature assessing the costs, and the costs of expanding, STI programmes. Specifically, it provides cost-estimates for an outreach and STI treatment programme targeting youths in Tajikistan. It uses costs observed in three pilot sites to construct a cost projection for expanding the programme. Further, it assesses possible sources of financing for the programme.

## Methods

### Setting

Starting in 2006, a programme encompassing outreach, confidential and voluntary counselling and testing, and STI diagnosis and treatment for most at risk adolescents (MARA) was piloted at three sites in Tajikistan. This programme was given technical and financial support by UNICEF, and implemented by the Tajik Association of Specialist in Dermatology and Venereal Diseases, Population Services International, and the public health services (all of which also provided some financial support), with support from other Tajik NGOs and government agencies. The pilot included regular outreach to MARA, with potential clients invited to newly established clinics (youth friendly health services (YFHS)) for confidential counselling, diagnosis and treatment of STIs. Diagnosis and treatment were offered free of charge, and condoms were distributed free of charge both during outreach and at the clinics. There are four main groups of most at-risk adolescents (MARA) in Tajikistan: sex workers, intravenous drug users (IDUs), men who have sex with men, and others (mainly street children) [11].

The overall prevalence of HIV remains low in Tajikistan; it is estimated that less than 1% of the population is HIV positive [12]. However, high-risk populations show a much higher HIV infection prevalence, with one 2004 report showing a 12% HIV prevalence among IDUs in Dushanbe [13]. Government reports indicate the HIV prevalence in Dushanbe and Khujand has reached as high as 26% among IDUs [14]. Further, the prevalence of other STIs is also high among sex workers with the prevalence of syphilis estimated at 12% [11], while among youth tested at pilot youth friendly health services, 39% were diagnosed with an STI [11].

General population knowledge about HIV/AIDS is low in Tajikistan. The 2005 Multiple Indicator Cluster Survey (MICS) showed only about 41% of the Tajik population had heard of AIDS, and only about 23% of respondents aged 15-19 years had heard of AIDS [15]. However, a 2006 survey of school children aged 13-15 years showed that just under half of respondents reported being taught about HIV in school, indicating that knowledge of HIV/AIDS, while still low, is higher for children coming-of-age over the next 5 years [16].

Among those that had heard of AIDS, about 32% of respondents in the MICS survey could identify one method of preventing HIV transmission, and only 11% could name three prevention methods (16% and 5%, respectively, among respondents aged 15-19 years) [15]. Among the 13-15 year old respondents of the school-based survey, less than 4% correctly answered five questions about HIV transmission vectors [16]. Thus, while knowledge of the existence of HIV is low, knowledge about preventative measures is very low, and especially low among adolescents. While data on sexual activity are scarce, among the youths aged 13-15 years in the school based survey, 13% reported having had sex in the last 12 months (of which more than half report using a condom), indicating that youths engaging in sex is not uncommon in Tajikistan [16].

Just under 13% of the women surveyed in the MICS survey knew a place where they could get an HIV test; this level was only 5% among 15-19 year old respondents [15]. However, in the school-based survey, 37% of respondents reported being taught a place where they could get the HIV test [16].

In June 2007, Tajikistan started a pilot introduction of a "Basic Benefit Package" (BBP) of health services in four rayons. The BBP seeks to regulate health care recipients' out-of-pocket payments, which account for an estimated 70% of health care financing, by introducing prospectively determined formal co-payments for health services from secondary-level facilities. The BBP decree defines a 'package' of services that will be exempt from co-payments for attending patients based on patients' social status or disease. This free benefit package includes "Anonymous consultations about HIV/AIDS and STI" for the entire population [17].

However, since most new HIV infections occur before the age of 25, there remains a need for youth-targeted HIV prevention outreach services (and not just the treatment covered in the BBP), especially for MARA [1]. Youth-friendly facilities are also needed to enable MARA to access health services because youth under the legal age are not covered under the BBP. Further, many MARA, due to their situation, do not have official registration cards, which are necessary to gain the benefits of the BBP programme.

### Target population

It is assumed that the programme needs to be expanded to the major cities of Tajikistan. To determine the population in need where the programme is to expand, the total number of youth (age 15-25) was derived from city or rayon-specific population figures. Then, the likely percentage of youth that are MARA was estimated by applying the percentage of youth that are MARA from the three pilot areas to the new areas. The number of MARA in the three pilot areas was determined using surveys conducted after 6 months of programme operation, where the total number of attendances at the YFHS facilities were multiplied upward based on the percentage of MARA interviewed for the survey who had attended the YFHS [11]. The total number of MARA attending YFHS facilities and receiving specific types of services are then calculated based on facility records for the first year of operation in the three pilot sites. Thus, the total number of facilities in operation, the number in the target population, and the number of patients receiving specific types of services are calculated for all areas designated for programme scale-up.

In some cases during the pilot, service utilization rates were limited due to supply stock-outs. For example, outreach workers felt that more condoms could have been distributed if supplies had been available; in two sites, stock-out of STI diagnostic supplies was reported. In these cases, in addition to the estimates based on the observed data, the number of patients treated is also calculated assuming these supplies would be available. These results are presented in scenario analyses.

### Costing

The study uses the government health system perspective. The cost of the programme at the three pilot sites was determined based on a review of programme and financial records and interviews with the three programme managers. Both government and donor records were reviewed. Costs were collected for the period of January 2007-June 2008 and converted to annual equivalent quantities. All costs are presented as incremental to the existing health system infrastructure.

Costs are classified into four categories for the purposes of analysis:

1. Facility investment (start-up costs): Capital improvements made at facilities so that they can offer youth-friendly services to MARA. Costs in this category include the purchase of furniture, medical equipment, and laboratory equipment. Building and utility costs were not considered since most facilities in Tajikistan are under-utilized and needed space for this programme should be available at future sites.

2. National level programme costs: Cost of training (of medical staff), supervision, the design and printing of informational brochures, and monitoring and evaluation.

3. Facility level programme costs: The costs of the operation of the programme at the facility level include airing of media messages, establishing and coordinating relations with other members of the community, cost of outreach, cost of monitoring, and the hire of a programme manager.

4. Variable costs: These include the costs incurred due to patients seeking service at the YFHS facility and cover counselling costs, consulting fees for the doctor, HIV testing costs, STI diagnosis, and STI treatment costs. The costs of services which require referral outside of the YFHS clinic, including management and treatment of HIV/AIDS among those found positive for the virus, are not included in this analysis. Since programme records include the number of people contacted in outreach, but do not list the total number of outreach contacts made, costs for outreach were determined by finding the number of condoms and brochures distributed and dividing by the number of unique contacts.

Costs were calculated based on an ingredients approach, multiplying the quantity of goods needed by the unit price of each item, using Microsoft Excel [18]. Annualized costs for goods with a lifespan of more than a year were calculated using a 10% discount rate, and are reported separately from routine costs. When necessary, prices were converted to US dollars using official exchange rates reported by the International Monetary Fund [19]. All costs are reported for the year 2007.

### Financing

In Tajikistan, the Family Medicine Programme has the terms of reference to serve adolescents and was considered one likely source of financing for programme scale-up. To determine the adequacy of resources available in the Family Medicine Programme, a series of different scenarios are explored. All data were gained from the Tajikistan Ministry of Finance (MoF) and the Ministry of Health (MoH).

In the first scenario, MoF macroeconomic projections through 2010 are used to determine the size of the overall economy, and subsequently project the size of the government budget. The percentage of the government budget dedicated to health is also derived from MoF projections, or, when these are unavailable, using the percentage from the previous year (i.e., it is assumed that there will be no change in the health budget as a percentage of the total government budget).

To determine the amount of resources available for the YFHS programme, the percentage of the health budget devoted to the Family Medicine Programme is determined based on historical budgets from the Ministry of Health (MoH). This is projected into the future assuming the Family Medicine Programme retains a constant percentage of the MoH's total budget. The amount of the Family Medicine Programme intended for adolescents is determined based on the assumed utilization rates of different age groups. However, these data are not available for each of the areas intended for scale-up, so specific figures are not available. Another potential source of financing comes from the STI programme, and further scenarios are developed based on data available for the STI budget at one of the pilot sites.

## Results

### Target population

The target population used for the costing estimates is shown in table 1. In total, eight sites were included, covering an estimated 8,020 MARA. The eight sites are located in cities that have a combined total population (all ages) of over 2 million, representing about 1/3^rd ^of the population of Tajikistan, and encompass almost 294,000 youths. Since the number of MARA is projected based on survey data from the three pilot sites, ranges of the number of MARA were also estimated based on the observed high and low percentage of youth being MARA; these high and low estimates are considered in scenario analysis (see below). The estimated range of MARA covered is 4,906 to 10,507.

**Table 1:** Estimated population in need and service utilization rates

Category	Estimated/observed in 3 pilot sites	Projected for 8 national sites	Ranges used in scenario analysis	
			
			Low	High
Number of Youth	163,945	294,469		

Number of MARA	4,068	8,020	4,906	10,507

Number of Facilities	3	8	8	8

Number of visits to YFHS per MARA per Year	0.602	0.602	0.602	0.800

Number of STI tests per YFHS visit	0.779	0.779	0.779	0.95

Number of HIV tests per YFHS visit	0.245	0.245	0.200	0.300

Number of STI infections treated per STI test	0.229	0.229	0.229	0.229

YFHS: Youth Friendly Health Services

MARA: Most at-risk adolescents

STI: Sexually Transmitted Infections

Service utilization rates are also shown in table 1, in which all data collected from the pilot sites were converted to annual rates. In the three pilot projects observed, there were on average 0.6 visits per year per MARA, an average of 0.77 STI tests per clinic visit, and 0.25 HIV tests per clinic visit. Finally, the treatment rate for STIs was observed at 0.23 per STI test performed. Programme managers reported that the observed rates of STI testing were lower than would have occurred in a fully supplied programme, and that, given better quality of care or different hours of operation, more MARA would be able to attend the facility. Thus, increases in both rates were explored in scenario analysis.

### Costs

Tables 2 and 3 present the results of the costing analysis.

**Table 2:** Variable costs: Unit costs for HIV and STI testing and STI treatment

HIV Tests	Number	Unit Costs (US$)	Cost per Episode
Lab test - Rapid Test	1	0.87	0.87

Lab test - Elisa	10%	1.88	0.19

**Total for HIV Testing**			**1.05**

**STI Tests**	**Percentage observed in 3 pilot sites**	**Unit Costs** **(US$)**	**Weighted Cost**
*Test for gonorrhoea*	34%	3.20	1.07
*Test for syphilis*	28%	1.56	0.44
*Test for herpes*	0%	3.49	0.00
*Test for Chlamydia*	4%	3.20	0.11
*Pregnancy Test*	2%	0.10	0.00
*Urine Test*	2%	2.91	0.06
*Test for trichomoniasis*	31%	3.20	0.99
**Average Unit Costs**			**2.68**

**STI Treatment**	**Percentage observed in 3 pilot sites**	**Unit Costs** **(US$)**	**Weighted Cost**
*Treatment for gonorrhoea*	48%	7.43	3.53
*Treatment for syphilis*	5%	2.83	0.13
*Treatment for herpes*	0%	1.16	0.00
*Treatment for Chlamydia*	1%	7.65	0.11
*Treatment for trichomoniasis*	46%	0.31	0.14
**Average Unit Costs**			**3.91**

STI: Sexually transmitted infections

**Table 3:** Estimated financial cost of YFHS programme year 2008-2010

Description	Unit Price	Pilot -- Operational Costs	National Programme -- Operational Costs	National Programme -- Including Annualized Costs	% of total costs
*Capital Cost*					

Facility Start up (cost per facility)	16,789				

Number of Additional Facilities				5	

Total Costs for Capital Investment				3,293	12% (10% - 13%)
**Subtotal: Capital Costs**				**3,293**	**12%** **(10% - 13%)**

*Programme Costs*					

*For entire programme*					
Development of materials	145	145	145		
Number of Operating Facilities		3	8		

Supervision	1,488	4,465	11,907	11,907	9% (7% - 10%)
Training (start-up)	2,862	-	-	1,602	1% (1% - 1%)
Booklets	145	436	1,163	1,163	1% (1% - 1%)
Media	1,090	3,270	8,721	8,721	6% (5% - 7%)
Personnel	2,093	6,279	16,744	16,744	12% (10% - 14%)
Community Relations	1,265	3,794	10,116	10,116	7% (6% - 8%)
Outreach	3,258	9,774	26,065	26,065	19% (15% - 21%)
Monitoring and evaluation	58	174	465	465	0% (0% - 0%)
**Subtotal: Programme Costs**		**28,338**	**75,325**	**76,929**	**56%** **(45% - 62%)**

*Variable Costs*	**Unit Price**	**Pilot -- Operational Costs**	**Operational Costs**	**Annual Costs**	**% of total costs**

Outreach contacts	1.96	2064	4069		
Facility visits	4.42	2449	4828 (2954 - 8406)		
STI Tests	2.68	1882	3710 (2270 - 7985)		
HIV Tests	1.05	600	1183 (591 - 2522)		
STI Treatments	3.91	430	849 (519 - 1826)		

Total cost for outreach contact		4,046	7,977	7,977	5% (5% - 6%)
Total cost for facility visits		10,819	21,330 (13,049 -- 37,138)	21,330 (13,049 -- 37,138)	13% (11% - 22%)
Total cost of STI tests		5,052	9,961 (6,094 -- 21,437)	9,961 (6,094 -- 21,437)	6% (5% - 13%)
Total cost of HIV tests		632	1,246 (622 -- 2,655)	1,246 (622 -- 2,655)	1% (1% - 2%)
Total cost of STI Treatments		1,683	3,318 (2,030 -- 7,140)	3,318 (2,030 -- 7,140)	2% (2% - 4%)

**Subtotal: Variable Costs**		**22,233**	**43,832**	**43,832**	32%

**Subtotal: Range for variable costs**			**(29,772 -- 76,347)**	**(29,772 -- 76,347)**	**(24% - 45%)**

TOTAL COSTS for programme	50,571	119,103	137,082		

RANGE: TOTAL COSTS for programme			(104,953 -- 151,528)	(123,022 -- 169,597)	

STI: Sexually Transmitted Infections

#### Facility investment (start-up costs)

In total, the capital investment in the pilot project averaged US$ 16,789 per site (US$ 3,293 annualized). Purchase of new medical equipment constituted over half of the total capital investment.

#### National level and facility level programme costs

Most of the national level programme costs are considered operational costs with the exception of training, which is considered a start-up cost. Supervision and the development of informational materials together are estimated to costs US$ 13,215 per year. The cost of running the programme at each facility is estimated at US$ 7,764 per year, for a total of US$ 62,112 per year. About 42% of the programme costs at the facility level are for running the outreach programme.

#### Variable costs

The unit costs of diagnosing and treating sexually transmitted infections (STIs) are detailed in table 2. Not shown in table 2 is the estimated cost per outreach contact. This is estimated to be US$ 1.96 for condoms and brochures per unique contact per year.

It is assumed for purposes of costing that the rapid test for syphilis would be procured and routinely used in the programme; unit costs reflect this assumption. The relative weight given to each disease is based on the experience of the three pilot sites. The average cost per person tested is estimated at US$ 2.68, while the average cost per STI case treated is estimated at US$ 3.91.

#### Total costs

Table 3 summarized the cost projections. Cost are presented for the 3 pilot sites, for the yearly operational costs of running the expanded programme (excluding capital and start-up costs), and including all costs, with capital and start-up costs included as the annual equivalent costs. Operational and variable cost when the programme is fully operational are projected to be US$ 119,159, ranging from US$ 104,953 to 151,524. Including annual equivalent cost for capital and start-up items raises this to US$ 137,082 (range: US$ 123,022 to 169,597).

Capital improvement at facilities represents 12% of the costs. Programme costs and facility level programme costs represent 56% of costs, and the costs for medical services, diagnosis, and treatment represent 32% of the costs (Table 3 presents the ranges for these percentages based on the scenarios).

### Financing

An analysis of the costs and the financing together is presented in table 4, table 5, and table 6. Table 4 shows the cost per recipient, per target population, and per capita. The annual cost (excluding capital costs) for the three pilot areas is estimated at US$ 12.43 per targeted MARA (and about US$ 0.04 per youth). This increases to US$ 14.86 per MARA (range US$ 14.37 to 21.28) in the projected scale up; the difference is due to the smaller target population per clinic, on average, in the non-pilot sites. Including the equivalent annual costs for capital items raises the cost per MARA to about US$ 17.09.

**Table 4:** Financial analysis of YFHS programme: Cost per person

	Observed in 3 pilot sites	For programme at full implementation	Range for programme	
			Low	High
*Number in Target Population*				
MARA	4,068	8,020	4,906	10,507
Youth	163,945	294,469		
Population (total)	1,303,594	2,024,457		
*Total Yearly Costs (does not include capital costs)*				
Estimate	50,571	119,159	104,953	151,528
*Yearly Costs (does not include capital costs)*				
Per MARA	12.43	14.86	21.28	14.37
Per Youth	0.31	0.40	0.35	0.51
Per Capita	0.04	0.06	0.05	0.07
*Yearly Costs (including annualized capital costs)*				
Per MARA		17.09	15.34	21.15
Per Youth		0.47	0.42	0.58
Per Capita		0.07	0.06	0.08

MARA: Most at-risk adolescents

**Table 5:** Financial analysis of YFHS programme: Initial analysis

	Budget in Dushanbe (2008)	Budget in Tursunzade (2008)	National Health Budget	Range	
				Low	High
*Current Budget for youth*					
Budget for Family Medicine Programme	2,627,903	589,814	18,814,620		
Budget for youth	228,839	124,605	7,134,332		
YFHS programme yearly costs (excluding capital) as percentage of youth budget	9.7%	15.8%	1.7%	1.7%	1.9%
YFHS yearly costs (excluding capital) as percentage of youth budget			1.9%		

*Realization rate from 2007 applied to budget for 2008 for Dushanbe and Tursunzade; 95% realization rate assumed for National Health Budget

YFHS: Youth Friendly Health Services

MARA: Most at-risk adolescents

**Table 6:** Financial analysis of YFHS programme: Alternatives for Tursunzade

*Scenario 1: Budget based on state standard per capita expenditure and number of youth visits*			Formula/notes
a. Youth aged 13-19	39,657	39,657	
b. State standard health budget per capita	0.37	2.32	$0.37 is state standard allocation; $2.32 is the annual budget for primary health care per capita in Tursunzade
c. Budget for youth according to standard	14,641	91,995	= a × b
Programme routine costs as percentage of youth budget	134%	21%	

***Scenario 2: Budget based on STI and dermatologic in-patient services***			**Formula/notes**

a. State standard budget for 1 bed-day	11.2		
b. Total annual budget for STI/dermatologic in-patient services (BBP)	21,717		From MoH records
Programme routine costs as percentage of STI budget	91%		

YFHS: Youth Friendly Health Services

MARA: Most at-risk adolescents

Table 5 compares the costs and the financing. The Family Medicine budget from Ministry of Health records was available for 2 of the 3 pilot sites (Dushanbe and Tursunzade), and the costs for these two pilot sites are compared to the budget in the first two columns. The two areas had a total Family Medicine budget of over US$ 3 million; it is estimated that about US$ 350,000 of this is allocated for youth. The cost of the pilot programme is estimated to be about 9.9% of this 'youth budget'.

The right hand side of the table uses national figures to assess the costs of the YFHS programme on a wider scale. Here, it is estimated that about US$ 7.1 million of the Family Medicine budget is targeted at youth. The YFHS programme costs represent 1.7% of that budget. However, the areas targeted for scale-up only cover about 1/3^rd ^of the population of Tajikistan, indicating that the YFHS programme could represent 5-6% of the 'youth budget' in the scale-up areas.

Finally, data were available in Tursunzade to do further analyses of possible financing sources for the YFHS programme. Table 6 provides details for two scenarios. In the first scenario, the budget for primary health care is analyzed. Two standards for cost per capita are used: US$ 0.37 (which is the national standard) and US$ 2.32 (which is the budget in Tursunzade). Based on the population and the standard costs per capita, a total budget for youth is estimated. The YFHS programme would represent 134% of the youth budget based on the national standard, but only 21% of the budget based on the Tursunzade standard. Similarly, Scenario 2 shows the YFHS programme budget as 91% of Tursunzade's STI programme budget.

## Discussion

The costing done for this report includes many assumptions. The most important is that the activities observed in the pilot reflect what is needed for a national programme. Based on data observed from the pilot projects, outreach comprises about 42% of the yearly costs of running the programme at the facility level. The exact nature of outreach activities needed, and that are effective, needs fuller assessment. The relatively short term nature of the pilot projects do not give adequate information to assess the extent to which outreach worker turnover will affect the quality of the programme and necessitate additional training sessions in the future. Further, quality staff at higher levels will be needed to engage in training and supervision of the programme staff to ensure that high quality implementation is maintained.

Capital costs are likely to be dependent on the existing situation in different sites for the YFHS. What is presented is a best first estimate; more detailed planning and budgeting will be needed as the project is expanded. The price of many of the goods was obtained from international agencies that have tax exempt status. It is not clear if the Ministry of Health is able to procure some or all of these goods tax-free or on such favourable terms as international agencies.

The population at risk, reached, and treated is also highly uncertain. The pilot sites represented the capital city and two cities near Tajikistan's borders, which may not reflect the situation in all areas of Tajikistan. This is dealt with by using different scenarios to estimate the total costs of the programme. More detailed decisions are needed on where YFHS targeting MARA will be located. The scenarios show a range of just under US$ 50,000 in annual costs (excluding capital costs) is possible. However, the extent of the costs are, at least somewhat, in the control of the programme. For example, ensuring adequate supplies will increase the likelihood that patients who need a diagnostic test will receive one. Ensuring adequate outreach and clinic hours will also likely mean that more of the target population accesses the clinic. Thus, poor logistics and support will lower the cost of the programme (*de facto *resulting in rationing and poor quality of care) while good logistics and support will result in greater costs (and also greater effectiveness).

The rates observed in the pilot project are likely to under-estimate the need for visits and testing in a fully operational programme, and potential ranges were estimated to explore the impact on the total costs of increasing the rates of visits and testing. The assumptions of increased utilization are contingent on a programme that will be able to provide more outreach and better service and thus marginally increase clients' demand for services. It is assumed that in a well funded programme there would be 0.8 clinic visits per MARA. This is quite a bit higher than the observed rate (0.6) and is intended to represent the maximum costs for the programme barring any major changes in how it is implemented.

The observed rate of testing patients for STIs was 0.77 per clinic visit; however, given the nature of the clientele, it is likely that close to all visits would require a test. Thus, the upper range is set at 0.95 tests per clinic visit. There were no reported stock-outs of HIV tests in the pilot projects; ranges are included only to explore the impact of patient uptake of voluntary testing on the total costs.

The relative number of test performed are based on the experiences of the three pilot project sites; however it is unlikely that a different mix of tests would substantially change the overall average cost for testing. The unit costs for treatment across the diseases, on the other hand, show more heterogeneity, with the treatment of gonorrhoea and Chlamydia being more expensive than other diseases. Thus, if the disease patterns experienced by the three pilot sites do not reflect the disease patterns on a national level, the cost of the programme would change.

The financing scenarios presented are incomplete. More work is needed to clarify the amount of money that is potentially available for financing YFHS, and potential sources for this financing. It is estimated that in the target areas, MARA represent 2.7% of the adolescent population. In areas where budgets are available (Dushanbe and Tursunzade), this programme represents about 10-15% of funds estimated to be available for adolescents through the Family Medicine programme. Clearly, MARA are in greater need of medical services than many adolescents, and their budget allocation and medical consumption should consequently be higher than that of the average adolescent. These extra costs need to be considered in the light of the fact that the cost-effectiveness of STI interventions is also highly favourable.

It should not be thought that the financing scenarios included in this report are the only means of financing this programme. For example, in the baseline case, the projections estimate that about 3,700 laboratory tests will need to be done. In the BPP pilot project, patients are charged a user fee for laboratory tests; raising this user fee to cover these 3,700 tests may be possible and represent only a small increase in the per-test charge. Further, some of the costs, such as capital investment or training, may be funded by donors, as was the case for the pilot projects. This study briefly assessed the Family Medicine and STI departments as potential sources of funding for the YFHS programme. It may also be the case that all (rather than just youth's 'fair share') of money in the Family Medicine budget would be available for the YFHS programme.

In the longer term, gains in efficiency, reallocation of funds away from inefficient programmes, and other means of redirecting funds may also be possible. However, it is recommended that the Ministry of Health and partners ensure that sustainable funding is available in the medium to long term for facility level activities and the procurement of pharmaceuticals and supplies. The best conclusion to be drawn from the analysis remains, thus, that more research is needed to understand the extent to which governmental budget money can be a source of funds for YFHS programmes.

Estimating the benefits of programmes is difficult, especially in the absence of direct knowledge about the effects of the programme. The results presented here should then be interpreted as conservative (i.e., high) estimates for the cost of the programme because they do not include any potential savings resulting from the decreased number of STI cases as a result of the programme. Specific types of preventative activities, which could influence the amount of services born by the health system, include:

1. The benefits of increased use of condoms [20]. An unknown percentage of MARA has STIs and do not get treated by the YFHS programme but may be contacted during outreach. In addition, condoms are distributed to all MARA seeking treatment at the YFHS. While condom usage rates (during the last sex act) among CSWs overall appears to be over 50% [21], it is not known to what extent this applies to MARA, or the effect the YFHS programme has had on their condom usage rates.

2. The benefits of other preventative activities (e.g., such as counselling, outreach messages, reduction in the number of unwanted pregnancies) [20]. Some of these benefits, such as reducing the spread of HIV due to increased condom use, likely have a small effect currently given the low prevalence rate of HIV in Tajikistan, but could have more substantial implications in the longer term.

3. Decrease in the transmission of STIs due to treatment. Successful and timely treatment for STIs should lessen, in many cases, the length of time for which MARA are infectious, and thus reduce the number of STI cases among the people having sex with MARA [22-26].

Lacking data on transmission dynamics specific to either Tajikistan or this programme, it is not possible to robustly assess the extent of these benefits. Informal analysis (available from the authors upon request) indicates it is likely that, in net, the programme will cost the MoH money, but the amount saved could be a considerable proportion of the amount spent. That this informal analysis did not show that this programme will, in net, be cost saving is due to the relatively large proportion of the YFHS costs borne at the programme level compared with the variable costs for diagnosing and treatment. This emphasizes the point made elsewhere that the full costs of a programme, rather than just the medical costs, need to be considered in estimating the costs as well as the cost-effectiveness of a programme [27,28].

## Conclusion

This study presents the costs and the potential sources of financing for scaling-up a programme from the pilot stage to an expanded level of coverage. It thus presents a practical financial analysis, following templates used at the global level [29]. While there is global evidence to show that the interventions analysed here are effective [1], the effectiveness of the programme in the context of Tajikistan is not known. There exists at the pilot level good data on utilization and treatment, but further work could be done to gain a better understanding of the impact of the programme. This could be done either with sentinel surveillance, or by tracking the level of STIs in MARA and other STI programmes over time. Additionally, the cost of the programme needs to be tracked over time to ensure that budget allocations meet the needs of the programme.

The costs per case produced by this report are of similar magnitude to international studies of the cost-effectiveness of STI treatment [2,3]. This may seem somewhat surprising since, for example, this study includes only the incremental, rather than total, costs of the programme. Thus, consultations fees for doctors are included, but their base salary is excluded, and much of the capital infrastructure used in the programme is not included in the costs. However, the concordance of findings does offer support, from the cost side, to the evidence base that outreach to and treatment of STIs in MARA is likely to be a very cost-effective intervention and that there are few programmes that will contribute more to disease aversion at a lower cost per case prevented.

## Competing interests

The authors declare that they have no competing interests.

## Authors' contributions

NK formulated the research questions, helped design the study, and led the data collection. BJ was responsible for data management and analysis, participated in the development of the methodology and drafted the manuscript. BS contributed to the development of the methodology, as well as data analysis and interpretation. All authors contributed to the writing and editing of the paper and approved the final version of the manuscript (BS approved of the final version in translation).

## Ethical approval

No ethical approval was needed for this study since there were no human subjects. All programme managers agreed to be interviewed before the interview took place. No data containing information that could identify individual patients were collected. Further, a written memorandum of understanding (MOU) between the Tajikistan Ministry of Health (MoH) and UNICEF was signed in regards to the conduct of project activity and this event was also planned and endorsed by MoH at the deputy ministerlevel in the JointAnnual Working Plan for 2008.
